# Analysis of the Thermal Properties of Soft Silica Limestone Walls of Traditional Buildings in Central Poland

**DOI:** 10.3390/ma18102399

**Published:** 2025-05-21

**Authors:** Aleksandra Gorączko, Paula Szczepaniak, Marcin Gorączko

**Affiliations:** Faculty of Civil and Environmental Engineering and Architecture, Bydgoszcz University of Science and Technology, Kaliskiego 7, 85-796 Bydgoszcz, Poland; paula.szczepaniak@pbs.edu.pl (P.S.); gorgon@pbs.edu.pl (M.G.)

**Keywords:** stone walls, insulation, soft limestone, opoka, regional architecture

## Abstract

The challenge of thermally upgrading traditional stone masonry buildings is addressed through the analysis of a representative example typical of regional rural architecture in central Poland, constructed using soft silica limestone and clay mortar. These buildings, which form an important part of the local cultural heritage, are increasingly becoming the subject of interdisciplinary research and conservation initiatives. This study presents a detailed characterization of the materials and architectural features specific to this building typology. Thermal transmittance calculations were performed and analyzed, with the use of THERM 7.6.1.0 software enabling precise modeling of the wall’s heterogeneous structure. The physical and thermal properties of natural materials—particularly soft silica limestone and clay—were taken into account. The analysis included evaluation of the heat transfer coefficient, temperature distribution, and heat flux density for a reference wall model, as well as for variants with both internal and external insulation layers. The study explores thermal comfort and energy performance within the broader context of preserving and reusing historic rural buildings. Furthermore, the findings are discussed in relation to current European energy efficiency regulations and heritage protection frameworks. The scientific value of this work lies in its context-specific, material-sensitive methodology and in providing practical insight into balancing energy retrofitting with architectural conservation.

## 1. Introduction

The pursuit of ensuring thermal comfort and reducing energy consumption for heating and cooling have recently led to a growing interest in the thermal modernization of vernacular, often historic, residential buildings constructed using outdated technical standards [1]. Improving thermal comfort through retrofitting is often essential to maintaining these buildings as habitable spaces. Such actions are supported or even required by various international regulations that emphasize energy efficiency and adaptation to current and anticipated climate changes [2].

The challenge of thermal modernization applies, among others, to stone masonry buildings. Stone was historically a popular construction material due to its local availability, durability, and aesthetic qualities, such as color, texture, and finish. Stone buildings are found across different climatic zones worldwide, and their extensive use within specific regions often led to the development of unique local craftsmanship and architectural styles. Traces of these traditions can still be seen today. Such structures are studied from historical, architectural, and sociological perspectives. Some studies address traditional masonry techniques and the principles of preserving authenticity during restoration work [3,4]. Others focus on the physical and mechanical properties of natural stone typical for specific regions, which are critical for understanding both structural and thermal behavior [5,6]. A separate group of contributions highlights the cultural and environmental value of stone architecture and the importance of documenting and maintaining regional identity through material and architectural expression [7,8,9]. Broader comparisons with contemporary construction approaches demonstrate the energy-relevant qualities of vernacular architecture in various climates [10]. Further research [11] investigates how principles of vernacular architecture can be applied in contemporary rural development, highlighting the balance between cultural continuity and functional modernization Vernacular stone architecture is also increasingly recognized for its environmental performance and is frequently protected as an important component of cultural heritage [12,13].

However, traditional stone buildings rarely meet modern energy efficiency standards, which makes a detailed analysis of insulation methods necessary for each case. The need for thermal modernization depends largely on the climatic zone and the type of stone and construction used. In some milder climates, hygrothermal analyses show that traditional solutions provide sufficient insulation and can be preserved in their original state or even replicated in modern construction [14,15]. Advances in computational methods, which consider the thermal inertia of massive structures [16,17], also support these findings. Still, most traditionally constructed buildings do not meet today’s standards, leading to numerous studies exploring retrofitting possibilities [18,19].

A common thermal modernization approach involves applying external insulation, which often covers the original stone façade. This creates a conflict between achieving technical standards and preserving the building’s original appearance. Additionally, regional and national regulations, especially in countries with a large number of historic buildings, often prohibit energy-efficiency measures that alter a building’s visual identity, proportions, or character [20]. Special provisions regarding the need to ensure the protection of cultural values during the thermal modernization of traditional buildings are also included in European-level documents [21,22]. In such cases, internal insulation often becomes the only viable solution to improve the energy performance. However, this method poses significant challenges, as it alters the hygrothermal behavior of the walls and changes boundary conditions [23,24,25,26], particularly in colder climates [26,27]. One example of such a change is the shift of the temperature gradient within the wall, which causes the dew point to move closer to the interior. As a result, the internal face of the original wall becomes colder and may remain within a temperature and humidity range that promotes moisture accumulation. This can lead to interstitial condensation at the insulation–wall interface, thereby increasing the risk of material degradation or mold growth. The use of vapor-permeable insulation materials may help reduce this risk by allowing gradual moisture transfer and drying, but it does not eliminate the need for careful hygrothermal assessment and proper indoor humidity control.

Analyzing the hygrothermal performance of stone masonry in vernacular and historic buildings presents many methodological difficulties. Case studies and meta-analyses [28,29,30] especially highlight challenges such as obtaining reliable material parameters, including density, composition, moisture content, and thermal properties. These issues are more pronounced for natural stone than for brick masonry. Moreover, accurately modeling wall structures is complex due to variations in thickness, material composition, and often heterogeneous structures. Additionally older buildings may also exhibit damage, material loss, or moisture issues. Standard calculations often assume monolithic walls, but the proportions of stone, mortar, and voids significantly impact results. Direct measurements frequently show discrepancies compared to analytical calculations [28,31,32].

Addressing the thermal performance of traditional stone masonry buildings presents a considerable scientific challenge due to the high regional variability in materials, construction techniques, and wall typologies. This diversity often necessitates localized, case-specific analyses, limiting the applicability of general models or assumptions. The present study contributes to bridging this knowledge gap by focusing on a representative example from central Poland, where soft silica limestone and clay were historically used in vernacular construction. These materials, particularly the lightweight and porous limestone, are difficult to characterize due to their heterogeneity and limited availability in modern laboratory datasets. By applying detailed hygrothermal simulations to this under-researched masonry system, the study expands knowledge of its thermal behavior under realistic boundary conditions.

The scientific and novel value of this work lies in its integrative and context-specific approach—combining in situ material characterization, simulation, and a comparative evaluation of retrofit strategies—to derive findings relevant for conservation, sustainability, and energy efficiency in heritage buildings. In particular, this study provides a practical comparison of internal and external insulation solutions, considering the architectural and physical limitations of small-scale traditional structures. The ultimate objective is to assess the thermal behavior of traditional stone masonry under realistic conditions and to evaluate the effectiveness and applicability of targeted energy retrofit measures, with implications for sustainability, building physics, and heritage conservation

## 2. Materials and Methods

### 2.1. Characterization of Vernacular Buildings

**Figure 1:**
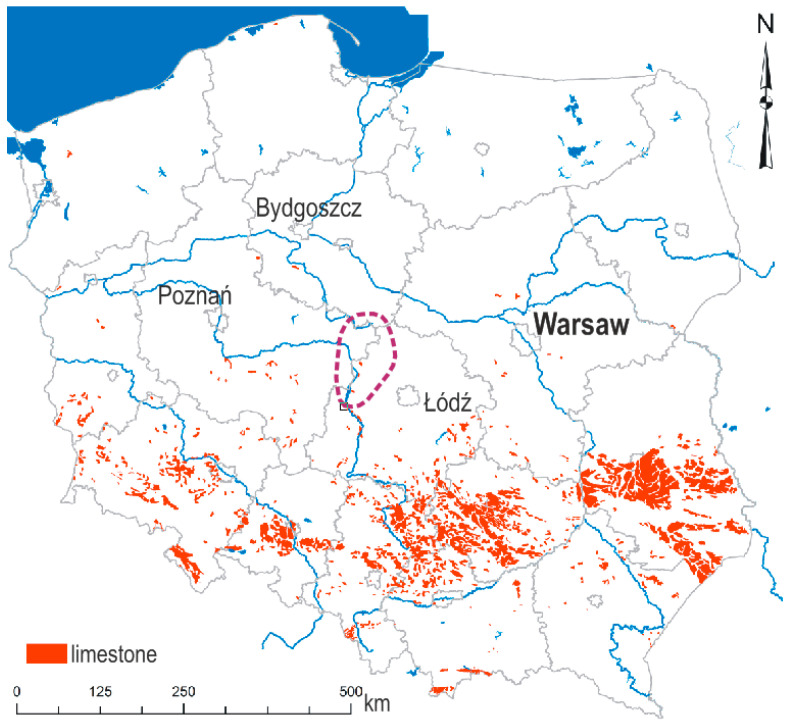
Map of Poland with limestone deposits and the distribution of described buildings (marked in the circle) in central Poland.

This study concerns traditional stone masonry structures in central Poland, built using soft silica limestone sourced from local quarries (Rożniatów, Poddębice, Poland). These buildings, numbering in the tens of thousands, were constructed in rural areas and small towns of this region from the late 19th century until the 1970s (Figure 1).

**Figure 2:**
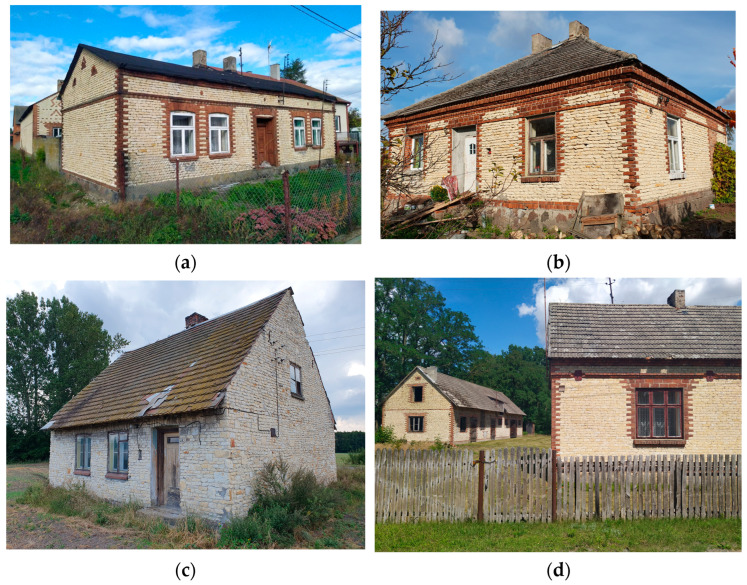
Examples of traditional buildings made of soft silica limestone in the analyzed area: (**a**–**c**) residential buildings; (**d**) farmstead.

During that period, a distinctive building tradition emerged in the vicinity of the quarries, shaped by the technology of stone wall construction [33]. These structures are mostly small, single-story residential buildings with simple architectural forms, although more prominent and representative buildings, such as churches and manor houses, can also be found (Figure 2).

Due to the widespread use of this construction technique, these buildings formed a characteristic architectural element of the region for many years. However, as a result of material degradation and incompatibility with modern technical and functional standards, this unique architecture is gradually disappearing. At the same time, it is increasingly recognized as an important cultural heritage and a source of inspiration [34]. Consequently, there is growing interest in a detailed assessment of their properties, including thermal performance.

### 2.2. Regional Method of Constructing Walls from Soft Silica Limestone

**Figure 3:**
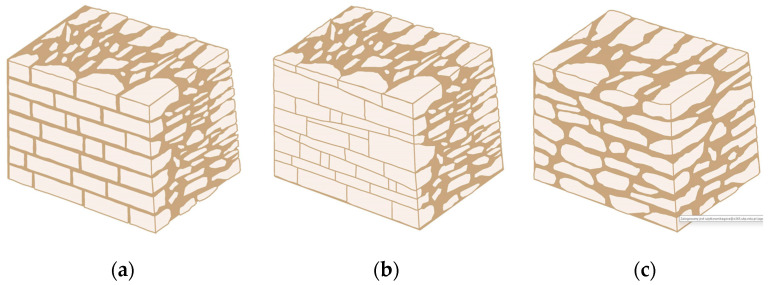
Typical layouts of traditional masonry walls made of soft silica limestone, representing the main types of masonry characteristic of the analyzed region: (**a**) regular wall (**b**) layered wall (**c**) wild wall.

Traditional walls made of soft silica limestone were typically supported by a shallowly embedded and slightly raised foundation wall constructed from split fieldstones and leveled with a layer of ceramic brick. The stone material delivered from the quarry was manually processed on-site. The raw material was easy to work with, and individual stone elements could be shaped with varying degrees of precision depending on their placement within the wall. It was very rare for the walls to be intentionally designed for external plastering. Instead, the stone façade was meant to remain exposed, which is why the stone elements were generally processed with great care. The most meticulously worked elements, shaped by splitting and trimming, were those intended for the facing surface of the wall (Figure 3).

The characteristic masonry texture is regular masonry (Figure 3a), a specific type of layered masonry (Figure 3b). Other solutions, such as mosaic or wild masonry (Figure 3c), were also used, though less frequently [35]. The layered and regular stone masonry, where all layers are horizontal and their height corresponds to that of a brick, represents the most recognizable architectural feature of buildings in this region. Ceramic bricks were used into the stone masonry at key structural points such as building corners, window and door frames, and cornice. The stone elements were bonded with clay mortar, made from natural clay extracted from the ground near the construction site. The joint width ranged from a few millimeters to 1 cm. The careful processing of stone elements primarily concerned the representative façades, whereas on the interior side, stone elements were laid in layers but not as rigorously as on the exterior.

The natural slaty structure of the rock in the deposit allowed stone elements to be incorporated into the masonry even without any processing. The goal was not to achieve a perfectly smooth wall surface; on the contrary, irregularities were deliberately left to enhance the adhesion of the internal clay plaster, which was applied in layers several centimeters thick. In cross-section, an infill layer was present between the external and internal layers of the wall (Figure 3). This core consisted of homogenized and plasticized natural clay mixed with smaller stone fragments and spalls, often waste material from processing the masonry elements. These fragments were arranged randomly or in a layered manner. The stability of the wall was ensured by the use of through-stones—long stone elements that alternately connected the outer and inner stone layers with the clay-stone infill.

According to historical building codes and guidelines, the typical and recommended thickness of soft silica limestone walls in residential buildings ranged from 50 to 60 cm [36]. Thicker walls were used for large-scale structures, such as barns and churches, whereas in farm buildings, the wall thickness was typically 40–50 cm.

### 2.3. Physical and Thermal Properties of Traditional Materials

#### 2.3.1. Stone Properties

Silica limestone from Rożniatów is a sedimentary rock with a characteristic light creamy or light yellowish hue, composed mostly of calcium carbonate and organogenic silica [37]. The rock is known by various regional names such as opoka (Poland and Lithuania), opuka (Czech Republic), Pläner (Germany) or gaize (France). The composition is predominantly calcite, representing accounting for approximately 70%. Laboratory tests have confirmed that the mechanical properties of the limestone are entirely sufficient for constructing residential and farm buildings. Its strength ranges from 13 to 16 MPa [34]. The values of the basic physical properties determined for the rocks from the primary extraction site (Rożniatów) are provided in Table 1.

Soft silica limestone from Rożniatów is a rock with a relatively low apparent density and high total (n_t_ = 45.3%) and open porosity (n_e_ = 42.9%) [37]. Connected porosity, measured using mercury intrusion, yielded a value (n_c_ = 34.23%), which is higher than water absorption at atmospheric pressure (A_b_ = 27.6%) but lower than the total porosity and even lower than the open porosity. The most probable cause is the impenetrability of the finest pores to mercury intrusion (<5 nm). Additionally, large pores could not be measured (>200 μm) [38]. Thus, soft silica limestone from Rożniatów has a lower density than typical ceramic brick but a significantly higher open porosity.

In the current standard PN-EN ISO 10456:2009 [40], which provides thermal conductivity (λ) values, data is available only for very soft limestones with a density of 1600 kg/m^3^. There is a lack of information for lighter and more porous rocks, such as tested soft silica limestone, which has a density of approximately 1420 kg/m^3^. Therefore, archival literature data [41] was used, according to which the thermal conductivity of Rożniatów limestone is λ = 0.53 kcal/(m·h·°C), equivalent to 0.62 W/(m·K). Similar values for highly porous rocks are provided in the archival Polish standard related to thermal protection issues [42] (Table 2). This standard differentiates limestones properties based on their bulk density and moisture conditions. This classification aligns with studies showing that the thermal conductivity of porous materials increases significantly with rising moisture content [43,44].

#### 2.3.2. Clay Mortar Properties

The clay material used as a masonry mortar, wall infill, and internal plaster is also of local origin. It is primarily composed of glacial clays, often (according to local interviews) lightened with the addition of sand and, in the case of internal plasters, sometimes with straw or sawdust. The granulometric composition [45] indicates a predominant content of sandy fractions and approximately 30% silt and clay fraction. And occasionally, a small amount of calcium carbonate (up to 5%) was present. The bulk density of clay mortars, according to our own research, is 1850 kg/m^3^.

The thermal conductivity value for clay soils according to the standard [40] is λ = 1.5 W/(m·K). Significantly lower values of λ = 0.7 W/(m·K) for sandy clays with a density of 1800 kg/m^3^ are provided by the standard [42], for both moderately humid and humid conditions. Meanwhile, in a more recent publication [46], the range of values for clay materials is given from 0.4 W/(m·K) (for lightweight clay plasters with fillers, e.g., microfibers) to 0.8 W/(m·K). Finally, the archival Polish building standard [47] was used, which provides the conductivity coefficient for clay masses used in construction from raw clay, depending on the bulk density (Table 3).

### 2.4. Assumptions of the Model and Calculation Variants

The reliability of numerical analyses using modern tools depends on the correct adoption of appropriate input data. In the previous subsections, the internal structure of the wall was presented, along with explanations of the basis for selecting thermal conductivity coefficients for the wall’s constituent materials. The lack of data on moisture-related parameters (diffusion resistance coefficient) for soft silica limestone led to the decision to perform only heat flow analyses. However, the undeniable influence of changes in the moisture content of masonry materials was accounted for only indirectly. When defining the calculation cases, likely moisture-change processes and the associated changes in thermal conductivity values were assumed.

The analyses were performed using THERM 7.6.1.0 software, which allows the calculation of two-dimensional steady-state heat flow with a non-orthogonal mesh grid. This enables the modeling of the complex wall structure, distinguishing between its components—stone masonry elements, mortar, and infill.

**Figure 4:**
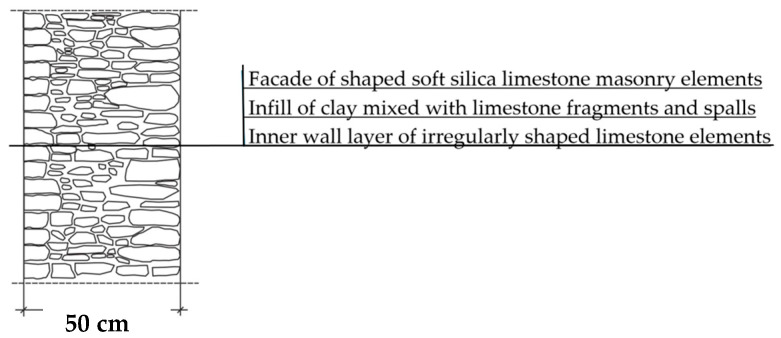
Basic geometric model of the wall for calculations in Therm software.

A geometric model of the wall was created, representing a typical masonry of the analyzed structures. The model assumes a wall thickness of 50 cm and dimensions reflecting the average real elements in the stone masonry while maintaining the layered structure and the proportions between the stone material and clay (Figure 4).

Heat transfer coefficient (U) calculations were carried out for six wall variants: three without insulation (A–C) and three with additional thermal insulation (D1, D2, E):

- Variant A: the basic model—a layered stone wall according to Figure 4, without plaster coatings. Possible moisture penetration of the external wall layer from atmospheric precipitation was assumed. For the internal layers, thermal parameters for stone and mortar in moderately humid conditions were adopted. It was assumed that the high open porosity of the stone and clay mortar allows for relatively free moisture movement and periodic drying of the walls. In the calculations, the surface resistance on the external and internal sides was taken according to standard [48], with values of R_se_ = 0.04 (m^2^∙K)/W and R_si_ = 1.13 (m^2^∙K)/W, respectively;
- Variant B: the version takes into account the commonly occurring internal clay plaster, typically 3.0 cm thick. Given the low vapor diffusion resistance reported for clay plasters ($\mu_{H_{2}O}$ = 6–10) [40,46], it was assumed that moisture flow was not significantly disrupted. The parameter values were adopted as those for the clay mortar used in the wall construction, as no significant differences in granulometric composition and density were identified [45];
- Variant C: in this variant, calculations were made considering the external cement plaster (1.5 cm thick). This is a situation commonly encountered in practice, typically applied when the outer wall layer begins to show signs of damage due to natural material wear over time or water exposure, leading to excessive water accumulation on parts of the façade. The thermal conductivity coefficient was adopted according to [37] λ = 1.0 W/(m·K). The cement plaster layer, on one hand, limits the periodic soaking of the external stone layer. Nevertheless, considering its higher diffusion resistance [45], it blocks moisture flow (especially in the case of insufficient ventilation) and may cause moisture accumulation in the partition. This was taken into account by assuming an increased value of λ for the stone and clay mortar elements, as in humid conditions;
- Variant D: the case with external insulation layer of polystyrene—the most commonly used variant in practice for thermal modernization. The calculations were performed for both the wall in a damp condition (variant D1) and in a moderately damp condition (variant D2), taking into account the phenomenon of gradual drying of the damp insulated wall over time [49];
- Variant E: the version with internal insulation, which allow the preservation of the original texture of the wall using high vapor-permeable panels, recommended for use in the renovation of historic buildings.

All parameters adopted for the calculations of the model, including geometry and boundary conditions, are presented in Table 4 (variants A–C) and Table 5 (variants D–E).

For the calculation of temperature distribution in the partition, the external temperature was assumed to be the long-term average based on the minimum daily temperatures for the winter months (December to February), which is approximately θ_e_ = −4 °C according to data from the Koło meteorological station [50], and the internal temperature θ_i_ = +20 °C.

## 3. Results

The results obtained from the THERM software for variants A-C without wall insulation are summarized in Table 6, while Table 7 presents the results for variants D-E, which include insulation.

The least favorable U-value of 1.19 W/(m^2^·K) was obtained for the wall in variant A without internal and external plaster. The application of a 3 cm internal clay plaster resulted in a slight decrease in the thermal transmittance coefficient to U = 1.14 W/(m^2^·K) (variant B). However, the additional application of an external cement plaster (variant C), assuming a reduction in the wall’s diffusivity leading to increased moisture content, had an adverse effect, causing the U-value to rise to 1.24 W/(m^2^·K). For each variant, the equivalent thermal conductivity coefficients (λ_equiv_) corresponding to a homogeneous wall were calculated, ranging between 0.57 and 0.62 W/(m·K).

The calculated surface temperatures on the interior side range from 13.5 °C for the damp wall with external plaster (variant C) to 13.7 °C for the wall with internal clay plaster (variant B).

The f_Rsi_ coefficient values range from f_Rsi_ = 0.73 to 0.74. This value is above the minimum hygiene criterion (0.72) according to [51]. This means that under normal usage conditions (i.e., residential spaces with standard humidity), the risk of condensation is low and may only occur in high-humidity areas (e.g., bathrooms, kitchens), especially during colder periods.

Due to the relatively similar thermal conductivity (λ) values of the individual wall components, i.e., soft silica limestone and clay mortar, which bind the elements and fill the inner part of the wall, the isotherms within the wall are very evenly distributed. According to the calculations, only the external layer of the wall, up to a thickness of approximately 10 cm, is exposed to low temperatures and cyclic freezing.

The heat flow rate slightly decreases after taking into account the internal clay plaster. However, the external cement plaster with low diffusivity, which, as assumed, can cause condensation of water vapor and moisture accumulation in stone and clay materials, results in an increase in the heat flow rate through the wall.

The results presented in Table 7 show that in the cases of external and internal insulation (Variant D2 and E), the calculated thermal transmittance (U-value) for the partition is U = 0.20 for 15 cm of insulation with a thermal conductivity of λ = 0.36. A slight difference appears in Variant D1, where a temporary increase in moisture content in the partition material after insulation was assumed, causing this parameter to rise to U = 0.21. The temperature distribution chart indicates that almost the entire thickness of the stone and clay wall is exposed to cyclic freezing.

## 4. Discussion

Calculations based on the actual wall structure and thermal transmittance coefficients (U) for variants A–C, which lack wall insulation, showed that the heat transfer coefficient was sufficient to meet the standards in place at the time of construction, i.e., in the 1960s, and remained compliant with Polish regulations until the 1980s [52]. However, it falls far short of current requirements specified in contemporary technical and building standards [51]. To meet these standards, a minimum insulation thickness of 15 cm is required to achieve the currently mandated U-value of 0.20, assuming the use of an insulation material with a thermal conductivity of λ = 0.36 W/(m·K).

In this context, the aspect of the significant thermal inertia of walls made of soft silica limestone should be addressed. Although Directive 2018/844 [21] does not specify recommended values for thermal inertia, it emphasizes its importance for energy efficiency. Thermal capacity does not directly affect the amount of heat loss through a partition under steady-state heat flow conditions. However, a high thermal mass provides the building with substantial thermal inertia, making the indoor environment resistant to short-term temperature fluctuations, which positively impacts the perception of thermal comfort throughout the year. It also plays a crucial role in maintaining the thermal stability of the indoor environment and can significantly reduce energy expenditures for heating and cooling the building.

The insulation variants considered in the calculations include the commonly used external insulation solution. However, this approach is highly destructive from the perspective of architectural heritage preservation, as it compromises the characteristic texture and color of the soft silica limestone masonry, which is typical for the region. The recommendations for the energy retrofit of traditional buildings in Directive 2018/844 [21] emphasize that the selection of energy efficiency measures should take into account the cultural value of the building, and that modernization efforts must not compromise the historical and architectural significance of the structure. Furthermore, when adapting buildings to contemporary energy standards, the directive recommends preserving original materials and construction techniques. This implies that for well-preserved stone facades, external insulation—despite being the most effective solution from a thermal modernization standpoint—should not be considered an acceptable option.

It is indicated that existing thermal insulation methods—if proper ventilation is ensured, preferably using diffusion-open insulation systems [21]—can protect the building from negative effects such as water vapor condensation. Nevertheless, a significant practical drawback in the case of the discussed buildings is the already limited internal volume, particularly the usable room height. Another concern with internal insulation is the unpredictable consequences of exposing the entire wall thickness to increased cyclic freezing, especially given the likely increase in wall moisture content.

In light of the above findings, the issue of improving the thermal insulation of traditional buildings made of soft silica limestone while preserving their architectural value is undoubtedly very complex, which places investors in a difficult decision-making situation. Directive 2023/1791 [22] emphasizes that EU Member States should support the education of their citizens and spread knowledge about the validity of renovation solutions, as well as provide transparent advisory systems in the field of investments aimed at improving the energy efficiency of buildings, especially in the context of the required improvement of buildings’ energy efficiency classes [2] one-stop shops for energy efficiency in buildings.

In Poland, for cases such as the one described, it would be advisable to implement a new tool supporting the energy renovation process in EU countries, namely ‘one-stop shops for energy efficiency in buildings’ [22] at the regional level. This would enable a more individualized assessment and selection of thermal insulation improvement options, tailored to the technical condition and cultural value of each building. The ongoing accelerated process of degradation of rural architectural heritage is also being observed in other regions of the country [53], leading to the development of interesting proposals for a comprehensive model of energy renovation management for traditional rural houses that takes national conditions into account.

## 5. Conclusions

This study presents a thermal analysis of traditional masonry constructed from soft silica limestone, characteristic of vernacular architecture in central Poland. Addressing a gap in current research, the work simulates the thermal behavior of this under-researched natural masonry material—soft silica limestone with clay mortar—considering both dry and moisture-affected thermal conductivity values. The results show that while uninsulated walls do not meet modern thermal performance standards under Polish climate conditions, their substantial thermal mass contributes significantly to indoor thermal stability. The study compares retrofit options and highlights the conflict between improving energy efficiency and preserving architectural heritage. Although external insulation provides superior thermal performance, it often compromises the appearance of historic façades. Internal insulation is visually less intrusive but constrained by limited interior space and the risk of moisture accumulation. Future research should focus on quantifying vapor permeability parameters and conducting long-term in situ monitoring to better evaluate retrofit outcomes.

These findings highlight the importance of retrofit strategies that are responsive to the specific physical behavior of traditional materials while respecting the cultural significance of heritage buildings. The results contribute to broader interdisciplinary discourse on enhancing thermal performance without compromising regional architectural identity.

## Figures and Tables

**Table 1:** Properties of soft silica limestone from Rożniatów—own research cited from [37,38].

Parameter	Unit	Mean Value	Min.–Max	Method
Apparent density (ρ_b_)	[kg/m^3^]	1420	1280–1550	EN 1936:2010 [39]
True density (ρ_r_)	[kg/m^3^]	2570	2520–2620	
Open porosity (n_e_)	[%]	43.9	41.4–47.3	
Total porosity (n_t_)	[%]	45.3	-	
Water absorption (A_b_) at atmospheric pressure	[%]	27.6	21.5-30.7	
Porosity (n_c_)	[%]	34.23	-	mercury intrusion porosimetry [38]

**Table 2:** Thermal conductivity (λ) values of porous limestones in the Polish standard [42].

Material	Bulk Density	Thermal Conductivity (λ) [W/(m·K)]	
	[kg/m^3^]	Moderately Humid Conditions ^1^	Humid Conditions ^2^
Porous limestone	1700	0.93	1.16
	1400	0.64	0.76

^1^ Moderately humid conditions—rooms with air humidity up to 75% during winter. ^2^ Humid conditions—rooms with air humidity above 75% during winter.

**Table 3:** Thermal conductivity (λ) values of clay materials in the Polish standard [47].

Material	Bulk Density	Thermal Conductivity (λ)	
	[kg/m^3^]	[Kcal/(m·h·°C)]	[W/(m·K)]
Heavy clay	1700–1800	0.70–0.80	0.81–0.93
Medium clay	1600–1700	0.40–0.70	0.46–0.81
Lightweight clay	1500–1600	0.20–0.40	0.23–0.46

**Table 4:** Comprehensive list of parameters assumed for thermal calculations in the THERM software for variants A–C (without insulation).

Variant					Parameter			
		External Surface Resistance (R_se_)		Thermal Conductivity (λ) ^1^				Internal Surface Resistance (R_si_)
			External Cement Plaster	Façade Limestone Elements	Clay Mortar and Clay Infill	Internal Limestone Elements	Internal Clay Plaster	
		(m^2^∙K)/W	W/(m∙K)	W/(m∙K)	W/(m∙K)	W/(m∙K)	W/(m∙K)	(m^2^∙K)/W
A	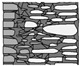	0.04	-	0.76	0.81	0.64	-	0.13
B	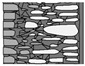		-	0.76	0.81	0.64	0.81	
C	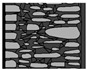		1.00	0.76	0.93	0.76	0.93	

^1^ Gray scale on the model proportional to the thermal conductivity (λ) value.

**Table 5:** Comprehensive list of parameters assumed for thermal calculations in THERM software for variants D–E (with insulation).

Variant		R_se_	Parameter							R_si_
			Thermal Conductivity (λ) ^1^							
			Polystyren	External Cement Plaster	Façade Limestone Elements	Clay Mortar and Clay Infill	Internal Limestone Elements	Internal Clay Plaster	Internal Porous Insulation	
		(m^2^∙K)/W	W/(m∙K)	W/(m∙K)	W/(m∙K)	W/(m∙K)	W/(m∙K)	W/(m∙K)	W/(m∙K)	(m^2^∙K)/W
D1	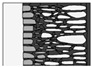	0.04	0.36	-	0.76	0.93	0.76	-	-	0.13
D2	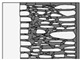		0.36	-	0.64	0.81	0.64	0.81	-	
E	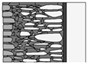		-	-	0.76	0.81	0.64	0.81	0.36	

^1^ Gray scale on the model proportional to the thermal conductivity (λ) value.

**Table 6:** Results of calculations in the THERM software for variants A–C (without insulation).

	Variant	Thermal Parameters	Isotherms	Temperature Profile	Heat Distribution	Flux Rate
A	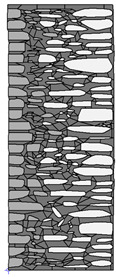	R = 0.84 (m^2^∙K)/W λ_equiv_ = 0.59 W/(m∙K) U =1.19 W/(m^2^∙K) θ_min_ = +13.6 °C f_Rsi_ = 0.73	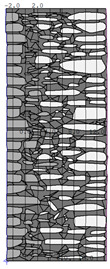	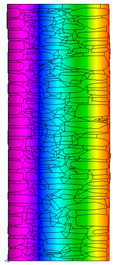	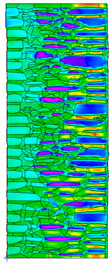	q = 24.03 ÷ 31.04 W/m^2^
B	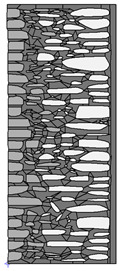	R = 0.88 (m^2^∙K)/W λ_equiv_ = 0.57 W/(m∙K) U = 1.14 W/(m^2^∙K) θ_min_ = +13.7 °C f_Rsi_ = 0.74	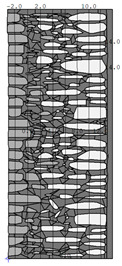	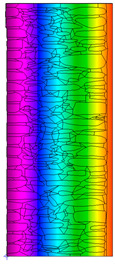	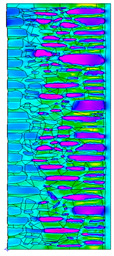	q = 23.03 ÷ 32.46 W/m^2^
C	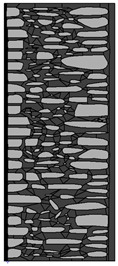	R = 0.81 (m^2^∙K)/W λ_equiv_ = 0.62 W/(m∙K) U = 1.24 W/(m^2^∙K) θ_min_ = +13.5 °C f_Rsi_ = 0.73	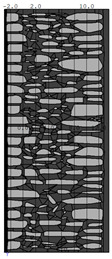	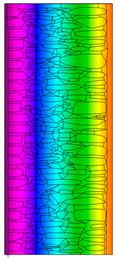	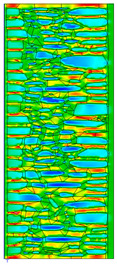	q = 25.45 ÷ 35.02 W/m^2^
				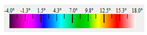	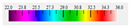	

**Table 7:** Results of thermal calculations in THERM software for variants D–E (with insulation).

	Variant	Thermal Parameters	Isotherms	Temperature Profile	Heat Distribution	Flux Rate
D1	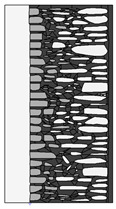	R = 4.84 (m^2^∙K)/W U = 0.21 W/(m^2^∙K) θ_min_ = +18.8 °C f_Rsi_ = 0.95	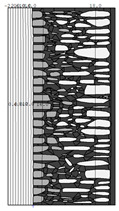	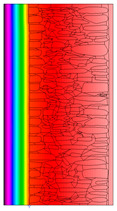	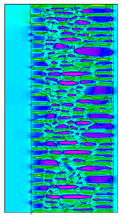	q = 3.67 ÷ 6.87 W/m^2^
D2	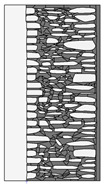	R = 4.90 (m^2^∙K)/W U = 0.20 W/(m^2^∙K) θ_min_ = +18.8 °C f_Rsi_ = 0.95	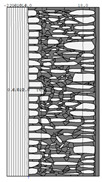	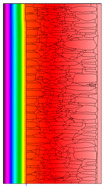	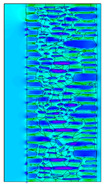	q = 3.68 ÷ 6.59 W/m^2^
E	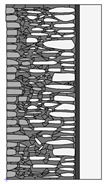	R = 4.90 (m^2^∙K)/W U = 0.20 W/(m^2^∙K) θ_min_ = +18.8 °C f_Rsi_ = 0.95	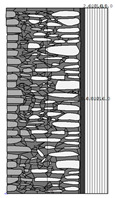	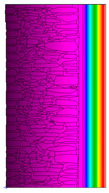	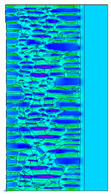	q = 4.08 ÷ 6.09 W/m^2^
				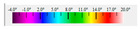	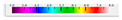	

## Data Availability

The original contributions presented in this study are included in the article. Further inquiries can be directed to the corresponding author.
